# Correction to “Aggressive surgery for incisional hernia with necrotizing soft tissue infection highlighting unique abdominal findings”

**DOI:** 10.1002/ams2.917

**Published:** 2023-12-26

Tokumaru T, Kurata H, Nakaebisu R, Tomioka J. Aggressive surgery for incisional hernia with necrotizing soft tissue infection highlighting unique abdominal findings. Acute Med Surg. 2023;10(1):e907.

In the third paragraph 3 of the text “the plate count, 20,200/μL” was incorrect. This should have read: “the plate count, 202,000/μL.

We would like to apologize for any inconvenience caused. Aside from this, there are no other errors, and the modifications do not change the overall structure or the central thesis of the paper.

